# Generation of apical-out nasal organoids to facilitate human respiratory syncytial virus infection and drug screening

**DOI:** 10.1016/j.isci.2026.116638

**Published:** 2026-07-02

**Authors:** Georgios Stroulios, Mathieu Hubert, Wing Chang, Allen Eaves, Sharon Louis, Philipp Kramer, Caroline Tapparel, Salvatore Simmini

**Affiliations:** 1 STEMCELL Technologies UK LTD, Cambridge, UK; 2 Department of Medicine and Molecular Microbiology, University of Geneva Medical School, Geneva, Switzerland; 3 STEMCELL Technologies Inc., Vancouver, BC, Canada; 4 Terry Fox Laboratory, BC Cancer, Vancouver, BC, Canada

**Keywords:** apical-out nasal organoids, 3D tissue culture, ECM-free organoids, culture temperature effect, respiratory syncytial virus, drug screening

## Abstract

The nasal epithelium is the first respiratory epithelium that is exposed to inhaled airborne pathogens. As a result, it is crucial to model host-pathogen interactions occurring in this tissue. To facilitate the efficient modeling of these interactions, we have developed a method to generate *de novo* apical-out nasal organoids from nasal epithelial cell aggregates. Optimization of this method revealed a stark tissue-specific effect of the culture temperature, as organoids were generated in much higher efficiency at 32.5°C compared to more widely used temperatures of 37°C. These organoids recapitulate the native tissue cellular composition of ciliated, basal, and goblet cells, while maintaining high homogeneity in size. Functionally, the system demonstrates susceptibility to viral infection and provides a robust platform for modeling antiviral drug responses. This standardized approach offers a reproducible system with high potential to be utilized in host-pathogen interaction studies and personalized medicine.

## Highlights

• Apical-out nasal organoids can be generated with a scalable and standardized method
• 32.5°C incubation optimizes generation efficiency in a tissue-specific manner
• Organoids are susceptible to RSV infection and can model antiviral drug efficacy

## Graphical abstract

**Figure:**
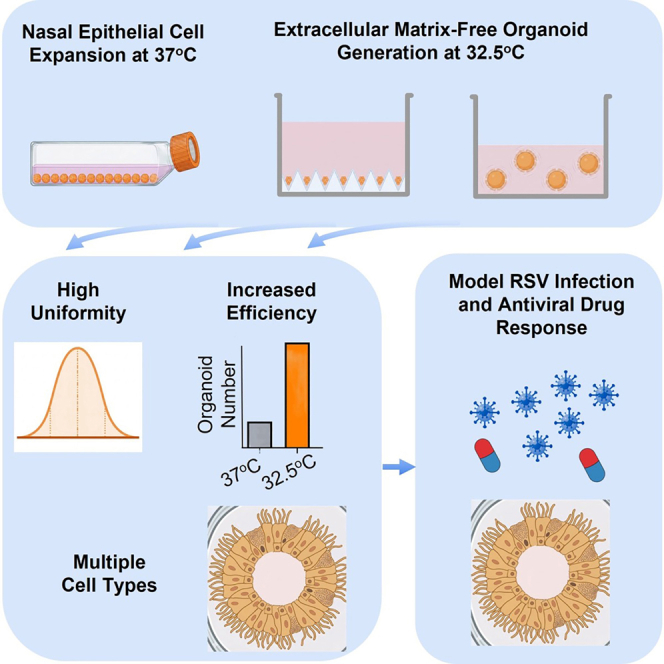

## Teaser

Biological sciences; Cell biology; Biological sciences research methodologies

## Introduction

The nasal cavities form a complex organ that exerts crucial functions, which include olfactory sensing, as well as filtering and conditioning inhaled air. The nasal mucosa lines most of the cavities’ luminal surface, with an epithelium composed of columnar and cuboidal cells attached to a basement membrane. The anatomical location of the nasal mucosa at the uppermost part of the respiratory tract renders it an attractive site for putative pathogens to enter the host.^1^ The exposure and susceptibility of the nasal epithelium to airborne pathogens present a substantial threat that can result in significant clinical burden.

A prime example of these pathogens is respiratory syncytial virus (RSV), an airborne virus of great public health concern, since nearly every child is infected by RSV already prior to age 2 and reinfections occur regularly throughout life.^2^ After acquisition, the virus mostly remains in the upper respiratory tract by infecting ciliated cells of the nasal epithelium but can progress to the lungs and cause severe lower respiratory tract infections (LRTIs) in immunocompromized patients, elderly individuals, young children, and neonates.^3^ To date, three vaccines (Arexvy, Abrysvo, and mRESVIA) and two monoclonal antibodies (palivizumab and nirsevimab) exist to prevent RSV infection in elder patients and children, respectively, and more than 20 vaccines are in clinical stage of development.^4^ In contrast, therapeutic options remain limited, with ribavirin as the only antiviral recommended to treat RSV-associated LRTIs, especially in immunocompromized patients.^5^^,^^6^^,^^7^

The nasal microenvironment plays a pivotal role in the entry and replication of RSV,^8^ but also several other airborne viruses such as human and mammal-adapted avian influenza A viruses^9^ or the severe acute respiratory syndrome-coronavirus 2 (SARS-CoV-2), the virus responsible for the recent coronavirus disease 2019 (COVID-19) pandemic.^10^ Since many more pathogens target the nasal mucosa, researchers have been eager to study the ontogeny and pathogenesis of infectious diseases in relevant model systems. To this end, a number of different *in vivo* models have been utilized to study host-pathogen interactions alongside the molecular mechanisms they employ. Yet, the presence of several anatomic and cellular interspecies differences, as well as the absence of crucial reflexes such as sneezing and coughing, have severely impacted the translatability of results to human disease.^11^

Numerous *in vitro* models allow for the manipulation and study of host-pathogen interactions, a critical step in understanding the mechanisms of respiratory illnesses. While simplified models, mostly based on immortalized cell lines, allow the study of specific disease aspects at the molecular level, they cannot recapitulate the tissue organization, systemic response, and mechanical forces that *in vivo* models can offer. Efforts are currently aiming at increasing the complexity of *in vitro* culture systems, in terms of both composition and structure, in order to maximize their physiological relevance.

*In vitro* culture of primary human nasal epithelial cells (hNECs) holds the potential to fulfill the need of increased complexity, and as a result researchers have focused on developing models based on this cellular source. However, expansion and differentiation of hNECs has been historically challenging, usually relying on a variety of both commercially proprietary and non-proprietary media optimized to culture human bronchial epithelial cells (hBECs).^12^^,^^13^^,^^14^^,^^15^^,^^16^ Different protocols have been developed to allow the generation of air-liquid interface (ALI) cultures in cell-culture inserts,^12^^,^^17^^,^^18^ which largely resemble the *in vivo* tissue regarding the cellular composition.^19^ Yet, the ALI culture system has inherent limitations, which include the limited scalability potential, a characteristic with paramount importance in the context of host-pathogen interaction studies and high-throughput drug screening.

Nasal 3D culture systems, such as organoids and spheroids, possess features to overcome some of these shortcomings. Nasal organoids have been successfully used to study cystic fibrosis,^20^^,^^21^^,^^22^ SARS-CoV-2 infection,^23^ and ciliary dyskinesia.^24^ Similarly to other epithelial organoid models, nasal organoid culture systems require the support of matrigel or similar extracellular matrix (ECM) hydrogels. This induces the stereotypical polarity conformation presented by epithelial organoids when embedded in an ECM hydrogel, where the apical surface of the epithelium faces inwards and is therefore not accessible from the external environment.

Recently, the generation of nasal 3D culture systems that expose the apical side of the epithelium to the environment has been described.^15^ These spheroids were derived from non-adherent epithelial sheets obtained from nasal brushings and have the potential to be utilized in host-pathogen interaction studies. However, they pose significant drawbacks including the rather limited scalability stemming from the absence of expansion capabilities and the use of undefined components, such as bovine pituitary extract or feeder extracts used in the medium.

Here, we optimized a workflow and culture conditions to generate a 3D apical-out nasal organoid (Ap-O NO) culture system and confirmed its susceptibility to RSV, as well as the capacity to detect antiviral drug effects. Similarly to the apical-out organoid model that we previously generated from bronchial epithelial cells,^25^ these Ap-O NOs demonstrate potential to overcome the shortcomings of previously described nasal cultures. Moreover, their polarity orientation offers the capacity to efficiently allow their use in host-pathogen interactions studies and other assays that require access to the apical surface of the nasal epithelium.

## Results

### Generation of Ap-O NO using the protocol optimized for airway apical out organoids

Given the biological similarities between hBECs and hNECs, we tested whether PneumaCult Apical-Out Airway Organoid Medium (AOAOM), a formulation previously optimized for the generation of apical-out airway organoids (Ap-O AO),^25^ had the potential to support the generation of Ap-O NO (Figure S1A). Single hNECs were aggregated in AggreWell 400 micropatterned plates to promote the generation of homogenous-sized Ap-O NO. Large numbers of similar-sized hNECs aggregates (Figure S1B) characterized by a dense, spherical morphology with minimal protrusions (Figure S1C) were efficiently established across multiple donors by day 15. However, at this stage a few aggregates survived and successfully differentiated into organoids with a dense central core and ciliated cells on the outer surface of the epithelium. Most organoids exhibited fragile and irregular morphologies with numerous shed single cells, indicating extensive cell (Figures S1D and S1E). The observed irregular morphology strongly suggested suboptimal culture conditions, as dense, spherical structures with minimal protrusions has been previously associated with successful and well-differentiated Ap-O AO.^25^ To benchmark the efficiency of Ap-O NO generation, we compared our results with historical data of Ap-O AO^25^ (Figure S1F). This analysis confirmed that Ap-O NO were generated with an efficiency of approximately 10%, much lower than the 60% efficiency observed in Ap-O AO derived from cells at the same passage. Fluorescent immunostaining analysis confirmed that these organoids were composed of acetylated α-TUBULIN-expressing ciliated cells, KERATIN5 (KRT5)-positive basal, and MUCIN 5AC (MUC5AC)-positive goblet cells (Figure S1G).

These results indicate that, similarly to the airway, functional nasal epithelial organoids can be generated with an apical-out configuration. Yet, the poor morphology and the rapid deterioration of the cultures, in addition to the high cell death, resulted in an overall low organoid yield that strongly indicated the requirement for optimization of the whole workflow.

### Lower culture temperature enhances the generation of human Ap-O NO: Protocol optimization

**Figure 1:**
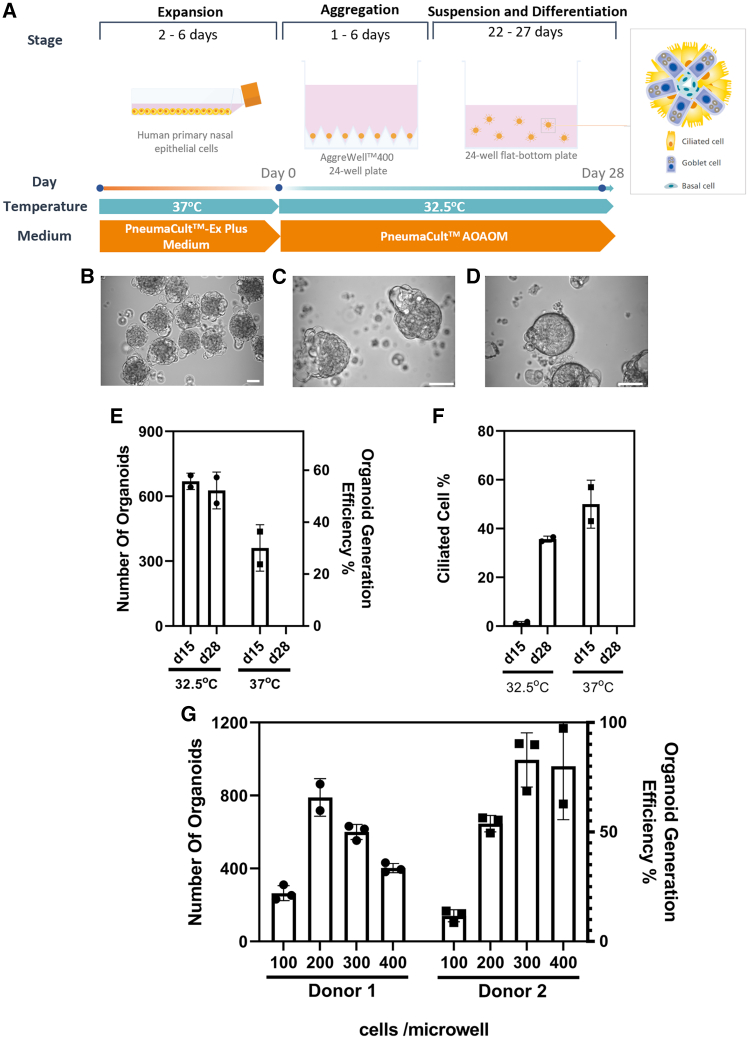
Lower culture temperature and higher seeding density improve Ap-O NO yield (A) Schematic of the modified protocol indicating the different temperatures used in each stage. (B–D) Representative brightfield image of organoids from 3 independent cultures from donor 1 (hNEC) generated at 32.5°C on day 1 (B), day 15 (C), and day 28 (D). (Scale bars, 50 μm). (E and F) Bar graphs comparing organoid number (E) and percentage of ciliated cells (F) in cultures generated at 32.5°C and 37°C at days 15 and 28, respectively. Bars indicate mean ± SD. Points represent technical replicates. (*n* = 1 donor). (G) Graph depicting the changes in organoid number and generation efficiency with modification of the seeding density in two donors at 32.5°C at day 28. Bars indicate mean ± SD. Points represent technical replicates. (*n* = 1 donor).

*In vivo*, the human nasal mucosal epithelium is exposed to a wide range of different internal and external environmental temperatures. In nasopharyngeal regions, temperatures were measured between 29°C and 37°C (average 32.6°C) with an ambient temperature of 23°C.^26^ Nasal epithelial temperatures were found to correlate with ambient temperatures, with cold air reducing epithelial temperature.^26^ Since cultures of different hNEC model systems are routinely generated and maintained at 37°C, we assessed whether different temperatures could influence the biological properties of nasal cells *in vitro.* To do so*,* we compared both the expansion and differentiation properties of cells cultured at 37°C and 32.5°C, which was selected as the temperature measured most frequently at the nasopharyngeal region.^26^ To assess temperature’s effect on differentiation, we first expanded all cells at 37^o^C. Subsequently, we initiated differentiation toward Ap-O NO at both 32.5^o^C and 37^o^C, using a protocol similar to the one established for airway organoids (Figure 1A). Aggregates were successfully generated at 32.5°C and when transferred into suspension culture exhibited a similar morphology to those observed at 37°C, characterized by dense, mostly circular structures with a defined border that occasionally displayed irregular protrusions on the outer surface (Figure 1B). This morphology was largely preserved in suspension cultures until day 15, although a few rare protruding bubble-like structures could be observed (Figure 1C). Importantly, at 32.5°C cultures could be maintained for at least 28 days (Figure 1D) with minimal morphological changes and cell shedding throughout the duration of the differentiation, in contrast to the high cell death and declining morphology observed in cultures at 37°C (Figures S1D and S1E). The differences in robustness were more apparent when we measured the generation efficiency of organoids derived from the two different temperatures (Figure 1E). Cultures maintained at 32.5°C demonstrated a higher organoid generation efficiency at day 15 when compared to those generated at 37°C (average organoid number per well of 669 at 32.5°C and 361 at 37°C). The improved capacity of organoids at 32.5°C to preserve their morphology for longer periods was also translated into higher organoid survival rates. Cultures at 32.5°C maintained the same number of organoids until day 28, by which time organoids generated at 37°C had completely dissociated (average organoid number per well of 627 at 32.5°C and 0 at 37°C). At that time point, organoids generated at 32.5°C displayed a halo of beating cilia on the outer surface (Video S1), a feature similar to that observed on day 15 in cultures generated at 37°C.

**Figure 2:**
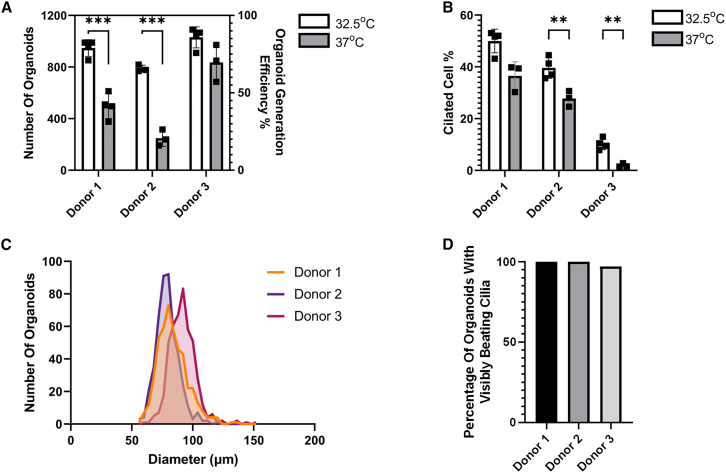
Modified workflow supports high yield, uniformity, and ciliary function in Ap-O NO (A and B) Comparison of the optimized and non-optimized protocols across three donors, showing quantification of organoid number (A) and ciliated cell percentage (B). Measurements were taken at day 28 for the optimized protocol and day 15 for the non-optimized. Bars indicate mean ± SD. Points represent technical replicates. Statistical significance was calculated using unpaired *t* test, with *p* < 0.05, ∗∗*p* < 0.01, ∗∗∗*p* < 0.001. (*n* = 3 hNEC donors). (C) Frequency distribution of Feret diameters for p4 organoids from three donors, based on measurements of at least 450 organoids per donor. (D) Percentage of organoids with active ciliary beating observed on the outer surface, assessed from 200 organoids per donor. (*n* = 3 hNEC donors).

Video S1. Representative video of Ap-O NO from hNEC donor 1 at day 28 with visibly beating cilia, related to Figure 2

To determine the differentiation potential of hNECs in these two temperatures, we compared the percentage of cells that displayed beating cilia on day 15 and day 28. On day 15, cultures at 37°C displayed robust ciliogenesis based on counts of ciliated cells (average 49%), whereas those at 32.5°C had few ciliated cells (average 1.4%). However, organoids maintained until day 28 at 32.5°C demonstrated much higher numbers of ciliated cells (average 35.1%) that more closely resemble physiological levels^27^ (Figure 1F and Table S1).

Finally, we optimized the workflow by testing different cell aggregation densities in AggreWell 400. We have previously described that 100 hBECs per microwell were sufficient to efficiently generate Ap-O AO and that higher seeding densities caused excessive cell shedding during suspension, resulting in organoids of similar size at the end of the workflow.^25^ To determine if phenotype and generation efficiency of Ap-O NO were also affected by the initial seeding density, we seeded 100, 200, 300, and 400 cells per microwell of an AggreWell 400 plate at 32.5°C. Interestingly, all tested seeding densities were able to generate Ap-O NO, but higher seeding densities demonstrated an increased organoid generation efficiency (Figure 1G). Organoid forming efficiency appeared to plateau between 200 and 300 cells seeded per microwell, with increasingly high seeding densities having a detrimental effect on the final organoid counts. As a result, we defined 300 cells per microwell as the optimal seeding density to be used for the efficient generation of Ap-O NO.

### Validation of the optimized workflow for robust generation of Ap-O NO

To evaluate our refined workflow, we derived Ap-O NO from 3 distinct donors and assessed several key aspects, including organoid generation efficiency, phenotype, and cellular composition. Considering the differences observed in organoids differentiated at 32.5°C, 37°C, and across different time points, characterization, and comparison analysis were performed at the time point where each temperature condition demonstrated its optimal phenotypic results. Therefore, we compared Ap-O NO generated at 37°C on day 15 (non-optimized workflow) to organoids generated at 32.5°C on day 28 (optimized workflow). The optimized workflow resulted in a significantly higher number of organoids and greater generation efficiency for all 3 hNEC donors, compared to the non-optimized workflow. For the optimized method, organoid counts averaged 949 (donor 1), 788 (donor 2), and 1,030 (donor 3), with corresponding organoid generation efficiencies of 79%, 65.6%, and 85.8%. In contrast, the non-optimized method yielded average organoid counts of 498 (donor 1), 248 (donor 2), and 835 (donor 3) and efficiencies of 41.5%, 20.6%, and 69.5%, with donors 1 and 2 displaying highly significant differences between the two conditions (Figure 2A). Similar morphological improvements were observed in cultures generated from either of the two new donors cultured at 32.5°C, when compared to 37°C. This indicated that the efficiency observed at lower temperature is an inherent characteristic of the modified approach, rather than stemming from a distinctive potential of the specific donor.

All Ap-O NO across conditions and donors were characterized by functional cilia that could be observed beating on the outer side, confirming the apical-out polarity. Quantification of the ciliated cell percentage indicated that the differentiation potential of Ap-O NO generated at 32.5°C was increased in all donors, with donors 2 (39.62%) and 3 (10.2%) showing a significant increase compared to what measured in cultures from the same donors at 37°C (donor 2 27.76%, donor 3 1.92%). Interestingly, donor 3 overall displayed a noticeably reduced population of cells with beating cilia compared to the other donors at both temperatures, highlighting the capacity of the model to preserve inter-donor heterogeneity *in vitro* (Figure 2B).

The use of the AggreWell platform to generate aggregates of homogeneous size has previously resulted in the establishment of Ap-O AO with a high degree of size homogeneity.^25^ To determine whether this characteristic was also preserved in Ap-O NO, we generated cultures using p3 hNECs and measured the Feret diameter of the resulting organoids (Figure 2C). This analysis demonstrated that Ap-O NO derived from all the tested donors displayed a remarkable size homogeneity, with an average diameter (± standard deviation) of 83.49 ± 12.2 μm, 79.16 ± 9.08 μm, and 91.37 ± 10.72 μm for donors 1, 2, and 3, respectively. The coefficient of variation was calculated as 14.63% for donor 1, 11.48% for donor 2, and 11.74% for donor 3, further highlighting the uniformity of the culture. This homogeneity was further confirmed by the quantification of ciliogenesis, as the vast majority of organoids (97%) derived from all 3 donors displayed visibly beating cilia at the outer surface (Figure 2D).

Since PneumaCult-Ex Plus could support the proliferation and serial passaging of hNECs, we tested whether these cells were also capable of retaining their differentiation potential at late passages. We harvested hNECs at the end of p4, the last passage they displayed proliferation, and used them to generate organoids using our optimized workflow. Even at the latest passage, the organoid generation efficiency remained above 50% across all donors, with each culture well capable of producing more than 600 organoids per AggreWell well of a 24 well plate (Figure S2A). When compared to the results obtained using cells of lower passages, the differentiation potential of high-passage hNECs seemed to be reduced (Figure S2B) but still remained within physiologically relevant levels.

**Figure 3:**
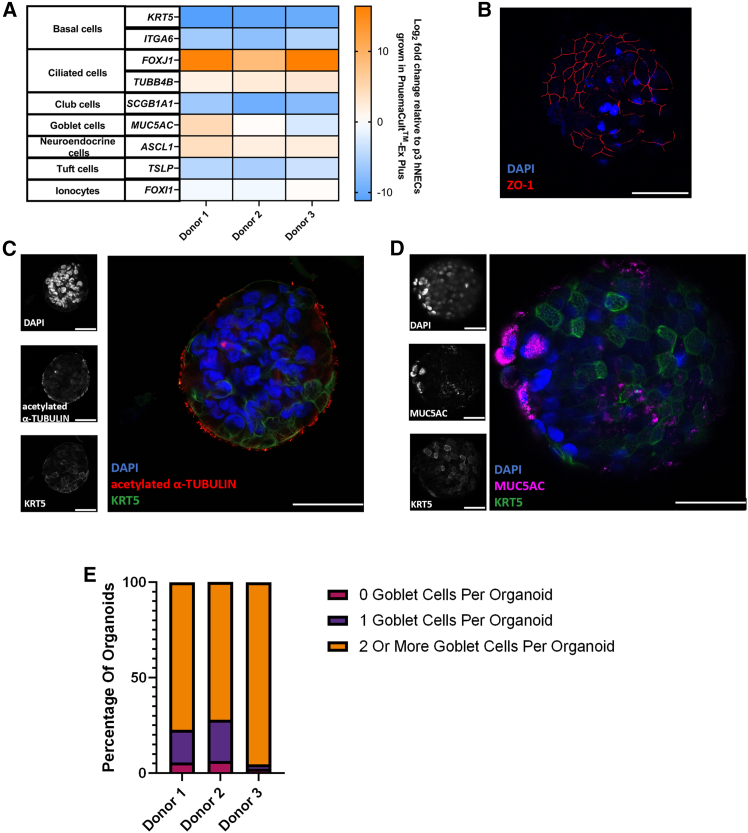
Successful differentiation and expression of key lung cell markers with the improved Ap-O NO workflow (A) Fold changes in the RNA levels of key lung differentiation markers in p4 hNEC-derived organoids from three hNEC donors. Expression was normalized to p4 hNECs cultured in PneumaCult-Ex Plus. (B–D) Immunofluorescence staining of organoids for the tight junction marker ZO-1 (red, B), the ciliary marker acetylated α-tubulin (red, C), the goblet cell marker MUC5AC (magenta, D), and the basal cell marker KRT5 (green, C, D). Nuclei are counterstained with DAPI (blue). Images are representative of three independent donors, showing hNEC donor 3. (Scale bars, 50 μm). (E) Stacked bar graph highlighting the percentage of organoids that contain 0, 1, and at least 2 goblet cells per organoid for each donor.

Finally, we characterized the cellular composition of Ap-O NO generated with our optimized protocol by harvesting them at day 28 and assessing the RNA levels of common markers of differentiated cell types (Figure 3A, Table 1). When compared to the RNA profile of hNECs cultured in PneumaCult-Ex Plus expansion medium, basal cell markers ITGA6 and KRT5 were significantly downregulated in Ap-O NO. In contrast, ciliated cell markers FOXJ1 and TUBB4B were significantly upregulated as a result of ciliated cell differentiation. Upregulation of goblet cell marker MUC5AC was also detected in two out of three donors. Presence of other secretory cells, such as club cells, could not be confirmed based on their marker (SCGB1A1) expression. Upregulation of markers characteristic of other rare cell types, such as tuft (TSLP) and ionocytes (FOXI1) were not detected. Interestingly, the neuroendocrine cell marker ASCL1 was found to be upregulated, albeit the relatively high Ct values (>36) do not allow for a definitive conclusion of the presence of this cell type. Immunocytochemical stains of fixed Ap-O NO detected a robust apical border on the outer surface, characterized by expression of tight junction protein ZO-1 (Figure 3B). Moreover, further analysis confirmed the presence of differentiated cell types. The ciliated cell marker acetylated α-TUBULIN was detected in all donors, with the localization of cilia on the outer surface further confirming the apical-out polarity of the organoids (Figure 3C). MUC5AC-positive goblet cells could also be readily detected (Figure 3D), as were KRT5-positive basal cells (Figures 3C and 3D). To better understand the frequency and distribution of goblet cells, we assessed the presence of MUC5AC-positive goblet cells (Figure 3E). Most (77.39%, 72.15%, and 95.41% for donors 1, 2, and 3, respectively) of the organoids contained at least 2 goblet cells, with a few (16.95%, 21.59%, and 2.29% for donors 1, 2, and 3, respectively) containing a single goblet cell. Ap-O NO devoid of goblet cells were rarely observed (5.65%, 6.25%, and 2.29% for donors 1, 2, and 3).

Collectively, these data validate our optimized workflow for supporting an efficient and robust differentiation of Ap-O NO from hNECs in an ECM-free manner across multiple donors. Such Ap-O NO contain multiple differentiated cell types representative of the nasal epithelium *in vivo* and display a high degree of size homogeneity, making them well suited for multiple downstream high throughput assays.

### Lower culture temperature impairs hNEC proliferation and delays differentiation

We next investigated the effect of temperature on hNEC proliferation by culturing hNECs derived from 2 donors in PneumaCult-Ex Plus expansion medium at either 37°C or 32.5°C. We then compared cellular morphology and population doublings (PD) across multiple passages. The morphology of hNECs cultures at 37°C was characterized by the presence of small, tightly packed cells in the first 3 passages (Figure S3A). A morphological decline was observed by p4, characterized by the formation of cell clumps with a “bubbly” appearance. Signs of stress were evident, with cells displaying an abnormally enlarged cytoplasm and vacuoles (Figure S3B), leading to growth arrest at p5. Similar to cultures grown at 37°C, cells cultured at 32.5°C exhibited a tightly packed, good morphology during the first passage that declined at day 9 when cultures ended (Figure S3C). Comparison analysis of the respective PD confirmed that hNECs cultured at 32.5°C were proliferating less (average PD until growth arrest 3.74) and slower (required 9 days to reach confluency), than cells cultured at 37°C (average PD until growth arrest 6.76 and required between 4 and 7 days to reach confluency) (Figure S3D). These results clearly indicated that temperatures lower than 37°C could not support an efficient expansion of hNECs *in vitro*.

Consistent with our observations that lower culture temperature slows organoid differentiation and nasal cell expansion, we investigated whether similar effects occur in ALI cultures. Compared to standard culture conditions (37°C), inserts differentiated at 32.5°C exhibited divergent morphologies (Figure S4A). While robust donors such as donor 1 were capable of establishing comparable morphology by day 21 at both temperatures, more sensitive donors (donors 2 and 3) generated thinner cultures characterized by prominent surface ridges at 32.5°C. As differentiation progressed to day 42, morphological shifts became more pronounced across all donors (Figure S4A, bottom row). At this time point, even the most robust donor (donor 1) exhibited significant morphological decline, while the remaining donors (donors 2 and 3) showed substantial epithelial retraction. However, the lack of apical leakage indicated that epithelial cells retained barrier function, which was further confirmed by high transepithelial electrical resistance (TEER) values (Figure S4B). Immunocytochemistry revealed a reduction in ciliated and goblet cell populations at 32.5°C compared to 37°C (Figure S2C). Notably, ciliary beating was absent at 32.5°C (Video S2), in stark contrast to the active beating observed at 37°C (Video S3). Extending the differentiation period to 42 days led to an increased presence of ciliated cells at 32.5°C (Figure S4C right column, Figure S4D) and allowed the detection of beating cilia (Video S4). However, differentiation of donor 2 was heavily impacted at both time points (Figures S4D and S4E). These findings suggest that while lower culture temperatures may modulate differentiation kinetics the effect is not universally beneficial, highlighting the necessity for platform-specific optimization.

Video S2. Representative video of a nasal ALI culture from hNEC donor 1 incubated at 32.5°C for 21 days, related to Figure 2

Video S3. Representative video of a nasal ALI culture from hNEC donor 1 incubated at 37°C for 21 days, related to Figure 2

Video S4. Representative video of a nasal ALI culture from hNEC donor 1 incubated at 32.5°C for 42 days, related to Figure 2

### Impact of culture temperature on Ap-O AO generation

To assess whether modifications related to the culture temperature were tissue specific, we tested if 32.5°C had any effect on the generation and morphology of Ap-O AO. After expansion of hBECs (*n* = 2 at p5) at 37°C, single cells were seeded in AggreWell 400 plates, at a density of 100 cells per microwell in PneumaCult AOAOM, either at 37°C or 32.5°C. At the end of the workflow on day 15, aggregates differentiated at 32.5°C in both donors had largely fused and formed one big aggregate of irregular shape (Figure S5A). These structures displayed protruding cells, suggesting the presence of extensive apoptosis (Figure S5B). Moreover, beating ciliated cells were not detected on the epithelial surface at this time point, implying that the lower temperature could have severely impacted the differentiation capacity of the hBECs. In contrast, cultures incubated at 37°C resulted in well-defined Ap-O AO and limited fusion, indicating the donor-specific optimization time to reduce fusion events was successful (Figure S5C). In addition, these organoids displayed the expected morphology, with a dense core and mostly spherical shape, with a clear darker border and minimal protrusions (Figure S5D). Ciliated cells could be readily identified beating, indicating the presence of robust differentiation.

Collectively, these observations suggest that the reduction in temperature, which had a paramount effect in the generation efficiency and morphology of Ap-O NO, had adverse effects in Ap-O AO generation. As a result, the beneficial effects of apical-out organoid generation at 32.5°C proved to be nasal specific.

### Functional assessment Of Ap-O NO for viral infection and antiviral drug testing

**Figure 4:**
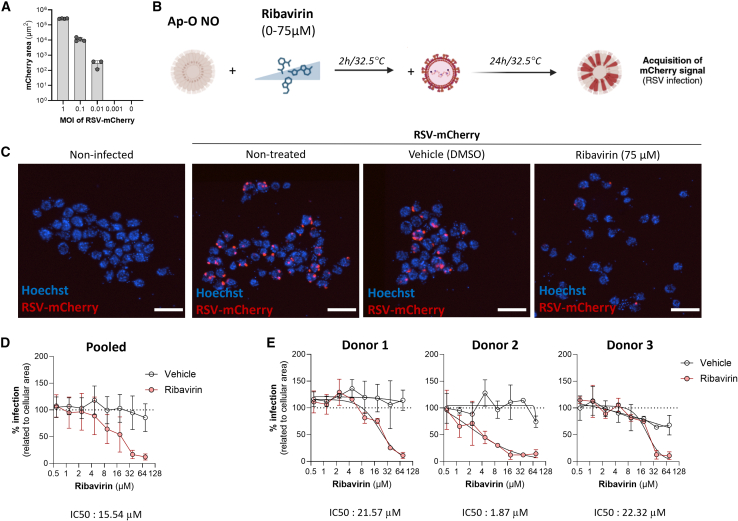
Ap-O NO are susceptible to RSV infection and are suitable for antiviral activity assessment (A) Representation of the dose-dependency of the mCherry area (expressed in μm^2^) in Ap-O NO in response to RSV-mCherry inoculum. Terminally differentiated Ap-O NO were exposed to RSV-mCherry at several multiplicities of infection (0.001–1) and the total mCherry area was measured as a surrogate of infection using a Cytation 5 plate imager. Data are presented as mean ± SD, with points representing techical replicates. (B) Terminally differentiated Ap-O NO were pre-treated with increasing concentrations of ribavirin (0–75 μg/mL) or vehicle (DMSO) alone. After incubation for 2 h at 32.5°C, treated Ap-NO were exposed to RSV-mCherry (MOI 0.1). At 24 h post-infection, mCherry signal was monitored by fluorescent microscopy as a surrogate of infection. (C) Representative examples of micrographs taken from non-infected and RSV-infected Ap-O NO (MOI 0.1) in the absence or the presence of 75 μM of ribavirin or vehicle alone. Nuclei are stained in blue (Hoechst) and RSV-infected cells are shown in red (mCherry). (*n* = hNEC 3 donors, scale bars, 200 μm). (D) Dose-response analysis of inhibition of RSV-mCherry (MOI 0.1) by ribavirin (red) or vehicle (white) in Ap-O NO collected from three independent donors. Data are presented as mean ± SD of the three individual donors. The IC50 value is indicated below the graph. (E) Individual dose-response analysis of each donor represented in D. Data are presented as mean ± SD of triplicates from one experiment. The IC50 values are indicated below each graph.

Similar to the airway, the nasal epithelium functions as a barrier to the outer environment and is targeted by a multitude of airborne pathogens. To test if Ap-O NO would be susceptible to viral infections and verify their potential to model these interactions, we infected terminally differentiated organoids with RSV. Exposing the organoids to increasing multiplicities of infection (MOI) of a recombinant RSV carrying an mCherry reporter (RSC-mCherry),^28^ allowed us to quantify the mCherry signal as a surrogate of infection. At 24 h post-infection (hpi), we observed a correlation between the viral inoculum and the RSV-mCherry area, which confirmed the susceptibility of Ap-O NO to RSV (Figure 4A). To test the capacity of the organoids to model antiviral drug effects, we next performed a dose-response assay with ribavirin, administered 2 h before infection (Figure 4B). Ap-O NO treated with 75 μM ribavirin showed markedly reduced mCherry signal compared to control, indicating a high antiviral efficacy in pre-treatment conditions (Figure 4C). A dose-depended effect of ribavirin was detected, with an IC50 of 15.54 μM (Figure 4D). Interestingly, the 3 different tested donors demonstrated varying IC50 values, ranging from 1 to 20 μM (Figure 4E). Taken together, these results show that Ap-O NO are sensitive to RSV infection and can be used for drug discovery.

## Discussion

Here, we describe a new method for the generation of Ap-O NO from primary human epithelial cells in the absence of any extracellular matrix. We detail the optimization of several aspects of the workflow to efficiently generate and differentiate Ap-O NO from 2D-expanded hNECs. Differentiated Ap-O NO were composed of ciliated cells that presented beating cilia on the outer surface of the organoid, as well as basal cells and mucin-producing goblet cells. Moreover, Ap-O NO displayed high size homogeneity and demonstrated great potential to be used in host-pathogen interaction studies based on their susceptibility to RSV and capacity to model the response to antiviral drugs like ribavirin.

The low organoid generation efficiency observed in the non-optimized workflow could be a result of insufficient growth factor supplementation, as media developed for the bronchial epithelia were used to culture hNECs. Despite the phenotypic, structural, and functional similarities shared by the two tissues, some differences might still exist at a molecular level. These could stem from the different germ layer of origin, as airway epithelial cells derive from the endoderm and nasal from the ectoderm.^29^^,^^30^ Differences have also been described at the expression level and activity of several key transcription factors between nasal and airway epithelial samples.^31^ Therefore, media optimized for other regions of the airway epithelium may inadequately represent the molecular niche of the nasal epithelium. For example, hNECs can be expanded in 2D using airway-specific media, however the colony forming efficiency drops dramatically after early passages.^32^ This is something that is also observed in expanding hBECs, albeit loss of proliferation capacity is observed at much later passages. This indicates that the current media formulations for the expansion of airway cells can be further optimized to better recapitulate the nasal stem cell niche found *in vivo*. Similar differences can also be observed in differentiation assays. Like hBECs, nasal cells can be differentiated in air-liquid interphase using porous membranes and commercially available airway media.^14^ However, the levels of ciliogenesis observed in these cultures supported by defined or undefined (containing bovine pituitary extract or calf serum) airway media formulations have been considered rather poor.^33^ Further characterization of the properties of the nasal epithelium both *in vitro* and *in vivo* will expand our knowledge regarding its physiology, resulting in the generation of novel *in vitro* models that more accurately recapitulate the *in vivo* tissue.

Interestingly, lowering the incubation temperature during differentiation improved the phenotype and generation efficiency of Ap-O NO. Extending the differentiation duration by two weeks promotes the development of well-differentiated Ap-O NO and optimizes the utilization of each donor’s cells, a significant advantage considering the relatively low expansion potential of hNECs *in vitro*. The presence of multiple differentiated cellular types indicates the functional resemblance of these organoids to the *in vivo* nasal epithelium. Moreover, the presence of beating cilia allows their utilization in the study of ciliopathies, as recently described for an Ap-O AO system.^34^

It has been shown that temperature also influences pathogen replication in nasal cavity: lower temperatures can enhance viral replication, while higher temperatures inhibit it.^35^^,^^36^ Considering this viral temperature sensitivity has been described to be exploited by the host’s immune system,^37^ it would be interesting to study its effects *in vitro*. Interestingly, similar inhibitory effects are also detected in mammalian cells, as culture temperature higher than that observed *in vivo* have been shown to constrain the proliferation of pluripotent cells.^38^ Moreover, temperature was described to influence the differentiation potential of keratinocytes,^39^^,^^40^ dermal cells that *in vivo* are exposed to similar temperature conditions as the nasal mucosa. Here, we showed that lowering the culture temperature to 32.5°C during differentiation drastically improved both the efficiency and morphology of the resulting Ap-O NO, across different passages. These might serve as an indication that culture conditions such as temperature should be assessed in a tissue- and assay-specific basis to supplement or augment the effect of different factors used to mimic the molecular niche.

*In vitro* nasal models have been employed to study the effect and infection route of several pathogens, including Influenza A,^9^^,^^41^ Rhinovirus 16,^42^ RSV,^14^^,^^43^^,^^44^ and SARS-CoV-2.^10^^,^^44^ Here, we demonstrated that Ap-O NO are suitable for RSV antiviral drug discovery, and their scalability potential has promising applications in high throughput screenings. Testing additional viruses will be needed to assess whether Ap-O NO are suited to study a large range of viruses, as observed with bronchial organoids.^25^ As a culture system, Ap-O NO present similar benefits to Ap-O AO, including a completely ECM-free and standardized workflow. However, the differences in the tissue accessibility offer additional advantages. Generation of both primary airway and nasal epithelial models requires a supply of donor cells, which have a finite lifespan. This results in a constant requirement for additional donors. Sourcing bronchial airway cells can be challenging, as they are routinely derived from patient materials, such as lavages, resections, and biopsies.^45^ Yet, such invasive procedures are not commonly undertaken, especially in healthy individuals. Moreover, the properties of the cells like the morphology, differentiation potential, and response to pathogens might be altered due to the individual’s disease background. Postmortem tissues are often presented as an option, although the potential impact that death had on the cells’ molecular mechanism and the effect of processing at different time points have not been assessed. As a potential alternative, nasal cells can be obtained from both adults and children, with minimal invasiveness through nasal brushing. This ease of sampling gives researchers the opportunity to recruit large datasets with desired characteristics (for example, age, sex, health status) to exclude potential bias arising from a small sampling group due to limited tissue availability. In addition, it allows the sequential sampling over multiple time points, enabling the study of phenomena in different age ranges, while maintaining the same genetic background. While this study relies on biopsy-derived primary cells, future evaluation of Ap-O NO and other nasal epithelial culture systems generated from nasal brushings could facilitate the widespread adoption of this highly accessible cell source. However, we think that researchers need to remain mindful of the differences between the nasal and airway epithelia to exclude potential generalizations.

The advantages of incorporating a diverse genetic background during drug screening were apparent in our testing, as different donors had alternate degrees of response to the same drug, ribavirin. Better understanding of the diverse drug actions that are influenced by the genetic background is something that is not easily feasible with immortalized cell lines due to their limited genetic diversity. Yet, organoid systems present not only an increased level of cellular and architectural complexity but also the ability to reveal demographic differences in drug efficacy. Recently, Ap-O AO were utilized in RSV antibody neutralization assays and described a cost-effective approach to not only detect neutralizing antibodies directed to the RSV fusion (F) protein like immortalized lines exclusively do but also those directed to glycoprotein (G).^46^ The characteristics of the Ap-O NO described here offer similar benefits and now present a valuable alternative to classical cell culture models. Furthermore, assessing the capacity of the system to capture the effect of different physiological tissue states (e.g., healthy, inflamed, chronic disease) has the potential to further increase its value. While the inclusion of *n* = 3 donors limits the extent to which our findings can be generalized to a larger and more heterogeneous population, future studies using larger and more diverse cohorts will be needed to further validate the broader applicability of these observed mechanisms. Leveraging the greater relevance of organoid systems and embracing the genetic differences between donors could lead to the development of treatments that are better tailored to an individual’s genetic background and a departure from generic treatments.

The scalability and uniformity of the apical-out culture system uniquely position it for applications requiring high throughput. A tight size distribution ensures that each organoid possesses a consistent surface area to volume ratio, which could promote uniform compound diffusion and support a reproducible number of cells across the entire screening plate. This structural homogeneity could minimize biological “noise” and statistical variance, enhancing the ability to distinguish true drug effects from random fluctuations. Compared to ALI, Ap-O NO present a more scalable and cost-effective alternative. Assuming equivalent cell numbers at the experimental endpoint, our Ap-O NO generation method reduces material costs by at least 4-fold compared to standard 96-well tissue culture inserts. This estimate is based on current list prices, targeting 100 organoids per tested compound at our reported mean generation efficiency. Notably, this estimate is conservative, as it does not account for potential efficiency gains associated with upscaling to larger 6-well AggreWell formats, reduced labor overhead for culture maintenance, or the implementation of more sensitive readouts that minimize the number of organoids required per condition. In comparison to other apical-out methodologies that result in a generation of a single organoid per well,^34^ our workflow offers clear upscaling benefits. These unique characteristics of the apical-out organoid culture system have allowed the use of Ap-O AO in high-throughput antiviral drug screening assays using 384-well plates.^47^ Ultimately, an optimal drug discovery pipeline would leverage the scalability of Ap-O NO for primary high-throughput screening, followed by the ALI model as a complementary approach for secondary hit confirmation.

However, inherent structural characteristics of the system may restrict its utility in specific experimental contexts. For instance, while the apical-out configuration of stochastically formed nasospheroids permits the modeling of CFTR function by monitoring shrinking upon activation,^15^^,^^48^ the suitability of Ap-O NO in these assays remains to be assessed. Furthermore, the submerged culture conditions prevent direct air exposure, complicating assays requiring aerosol delivery, and could result in the continuous dissolution of secreted mucus into the culture medium, thus inhibiting studies focused on mucus accumulation, rheology, or mucociliary transport. Additionally, while an apical-out polarity allows the modeling of apical-specific events like viral entry, it inherently restricts access to the basal surface. This structural limitation hinders studies requiring basal exposure, such as evaluating the basolateral delivery of antiviral compounds. Consequently, the selection of the optimal culture system based on the technical requirements of the downstream assays and the specific scientific question under investigation remains the best practice for researchers.

In summary, we have shown that Ap-O NO can be successfully generated using an ECM- and chemically defined medium and workflow, initially developed for the airway and subsequently optimized for nasal tissues. These organoids closely resemble functional, cellular, and architectural characteristics of the native nasal epithelium, exhibiting a high level of homogeneity and coordinated ciliary activity. Furthermore, their susceptibility to RSV infection and their dose-dependent response to ribavirin administration highlight their potential as platform for disease modeling and antiviral drug screening applications.

### Limitations of the study

The current study presents limitations, which we have attempted to highlight and discuss in the discussion section. Briefly, the small cohort of three donors tested, restricts the extent to which findings can be generalized to larger, more heterogeneous populations. Consequently, potential age- and sex-associated effects were not assessed. Architecturally, the submerged culture conditions prevent direct air exposure, complicating experiments that require aerosol delivery. This environment also leads to the continuous dissolution of secreted mucus into the medium, hindering research into mucus rheology, accumulation, and mucociliary transport. Furthermore, while the apical-out configuration facilitates access to the apical surface, it inherently restricts access to the basal side, limiting its utility for studies requiring basolateral drug delivery. Finally, the suitability of this specific organoid model for assessing CFTR functionality remains unverified.

## Resource availability

### Lead contact

Further information and requests for resources and reagents should be directed to and will be fulfilled by the lead contact, Salvatore Simmini (salvatore.simmini@stemcell.com).

### Materials availability

This study did not generate new unique reagents.

### Data and code availability

• Data reported in this paper will be shared by the lead contact upon request.
• This paper does not report original code.
• Any additional information required to reanalyze the data reported in this paper is available from the lead contact upon request.

## Acknowledgments

The authors thank the Cambridge Advanced Imaging Centre for their support and assistance in this work. This research was funded by the 10.13039/501100007601Horizon 2020, European Union grant OrganoVIR 812673 on the project ‘Organoids for Virus Research - An innovative training-ITN programme’. This research was also funded by STEMCELL Technologies Ltd., Cambridge UK and the Department of Medicine and Molecular Microbiology, University of Geneva Medical School, Geneva, Switzerland.

## Author contributions

G.S., M.H., C.T., and S.S. designed the study. G.S. and M.H. performed the experiments. A.E., P.K., S.L., and W.C., provided critical input and materials. S.S. and K.P. provided guidance. G.S., M.H., C.T., and S.S. wrote the manuscript with contributions from all the authors.

## Declaration of interests

G.S and S.S. are employees of STEMCELL Technologies Ltd., Cambridge, UK. A.E., S.L., W.C., and P.K. are employees of STEMCELL Technologies Inc., Vancouver, Canada. A.E. is the founder and CEO of STEMCELL Technologies Inc., Vancouver, Canada and STEMCELL Technologies Ltd., Cambridge, UK. G.S. and S.S. have provisional patent applications related to this research.

## Declaration of generative AI and AI-assisted technologies in the writing process

During the preparation of this work, the author(s) used Google’s Gemini in order to prepare the graphical abstract. After using this tool or service, the author(s) reviewed and edited the content as needed and take(s) full responsibility for the content of the publication.

## STAR★Methods

### Key resources table

**Table:** 

REAGENT or RESOURCE	SOURCE	IDENTIFIER
**Antibodies**		

Mouse Anti - ZO-1	Invitrogen	cat#: 339100; RRID: AB_2533147
Mouse Anti - acetylated TUBULIN	Sigma	cat#: T7451; RRID: AB_609894
Rabbit Anti - Keratin 5	Biolegend	cat#: 905501; RRID: AB_2565050
Mouse Anti - MUC5AC	Abcam	cat#: ab212636; RRID: AB_3083499
Goat anti-Mouse IgG2b Cross-Adsorbed Secondary Antibody, Alexa Fluor 568	Thermo Fisher Scientific	cat#: A-21144; RRID: AB_2535780
Goat anti-Mouse IgG1 Cross-Adsorbed Secondary Antibody, Alexa Fluor 488	Thermo Fisher Scientific	cat#: A-21121; RRID: AB_2535764
Donkey anti-Rabbit IgG (H + L), Alexa Fluor 647	Thermo Fisher Scientific	cat#: A-31573; RRID: AB_2536183

**Bacterial and virus strains**		

RSV-mCherry	Jean-François Eléouët, INRA, France	N/A

**Chemicals, peptides, and recombinant proteins**		

Ribavirin	Merck	cat# R9644
Triton X-100	Sigma Aldrich	cat# 10789704001
DMEM/F12	STEMCELL Technologies	cat# 36254
Tween-20	Sigma Aldrich	cat# P9416
TrypLE Express	Fisher Scientific	cat# 11558856
SunJin’s RapiClear 1.4	Scientific Laboratory Supplies	cat# RC149001
4′, 6-diamidino-2-phenylindole (DAPI)	Cayman Chemical	cat# 14285
Trypan Blue	STEMCELL Technologies	cat# 07050
Ambion™ DNase I	Invitrogen	cat# AM2222
SuperScript III	Invitrogen	cat # 18080-044
TaqMan™ Fast Universal PCR Master Mix (2X), no AmpErase™ UNG	Applied Biosystems	cat# 4352042
Heparin	STEMCELL Technologies	cat# 07980
PneumaCult^TM^-Ex Plus Medium	STEMCELL Technologies	cat# 05040
Animal Component-Free (ACF) Cell Dissociation Kit	STEMCELL Technologies	cat# 05426
Anti-Adherence Rinsing Solution	STEMCELL Technologies	cat# 07010
PneumaCult^TM^-ALI	STEMCELL Technologies	cat# 05001
PneumaCult^TM^ Apical-Out Airway Organoid medium	STEMCELL Technologies	cat# 100-0620

**Critical commercial assays**		

*KRT5*	Integrated DNA Technologies	cat# Hs.PT.58.14446018
*ITGA6*	Integrated DNA Technologies	cat# Hs.PT.58.453862
*FOXJ1*	Integrated DNA Technologies	cat# Hs.PT.58.40371261
*TUBB4B*	Integrated DNA Technologies	cat# Hs.PT.58.15334509.g
*SCGB1A1*	Integrated DNA Technologies	cat# Hs.PT.58.1190800
*MUC5AC*	Integrated DNA Technologies	cat# Hs.PT.58.24664713.g
*TSLP*	Integrated DNA Technologies	cat# Hs.PT.58.22464960
*FOXI1*	Integrated DNA Technologies	cat# Hs.PT.58.40514934
*TBP*	Integrated DNA Technologies	cat# Hs.PT.39a.22214825(1)
RNeasy Mini Kit	Qiagen	cat# 74106

**Experimental models: Cell lines**		

Primary human airway epithelial cells	EPITHELIX SARL	cat# EP51AB

**Software and algorithms**		

Graphpad Prism	GraphPad Software, Inc.	N/A
BioTek Gen5	Agilent Technologies, Inc	N/A
Leica Applications Suite X (LAS X)	Leica Microsystems	N/A
ImageJ	National Institutes of Health	N/A

**Other**		

24-well AggreWell^TM^ 400 plates	STEMCELL Technologies	cat# 34411
Costar® 6.5 mm Transwell®, 0.4 μm Pore Polyester Membrane inserts	STEMCELL Technologies	cat# 38024

### Experimental model and study participant details

Primary hNECs and hBECs used in the study are from biopsies of non-smoking, healthy donors and purchased from EPITHELIX SARL (cat # EP51AB). Three nasal and two bronchial donors were utilized across experiments, with the following demographics and characteristics:

• Donor 1 (hNEC): 37-year-old female of alternative geographic/ethnic origin (“Other”). (Lot #AB0857)
• Donor 2 (hNEC): 49-year-old Caucasian female (Lot #AB0869.02)
• Donor 3 (hNEC): 21-year-old Caucasian male. (Lot #AB0890)
• Donor 1 (hBEC): 62-year old Hispanic male. (Lot #02AB079301)
• Donor 2 (hBEC): 59-year old Caucasian male. (Lot #02AB0839.2)

### Method details

#### Expansion of primary human nasal epithelial cells

Primary hNECs used in the study are from biopsies of non-smoking, healthy donors and purchased from EPITHELIX SARL (cat # EP51AB). Initial cryopreserved hNECs were seeded as passage one (p1) into T25 cell culture tissue flasks in complete PneumaCult^TM^-Ex Plus Medium prepared as per the manufacturer’s instructions (STEMCELL Technologies, cat # 05040) and incubated at 37°C and 5% CO2 with a full medium change every second day. Once cells reached 60–70% confluency they were dissociated using the Animal Component-Free (ACF) Cell Dissociation Kit (STEMCELL Technologies, cat # 05426) following the manufacturer’s instructions.

To assess the effect of temperature on the expansion potential of hNECs, cryopreserved cells at p2 were thawed and expanded for one passage at 37°C. Upon reaching confluency, cells were collected and seeded in PneumaCult^TM^-Ex Plus either at 32.5°C or 37°C. Cells were serially passaged upon reaching confluency at each culture passage, until the cultures ceased expanding. Population Doublings (PD) were calculated using the following formula:

$$PD=3.322*\left(\log_{10}\left(number\;of\;cells\;harvested\;at\;the\;end\;of\;passage\right)-\log_{10}\left(number\;of\;cells\;seeded\right)\right)$$

#### Cultureware preparation

All plates used in organoid generation and in downstream assays were treated with Anti-Adherence Rinsing Solution (STEMCELL Technologies, cat # 07010) to reduce cell adhesion as previously described.^25^ For all types of 24-well plates used, 500 μL of Anti-Adherence Rinsing Solution were added to each well. After centrifugation for 10 min at 1300 g, Anti-Adherence Rinsing Solution was removed and each well was washed once with 1 mL DMEM/F12 (STEMCELL Technologies, cat # 36254). The wells were used directly after removing the wash medium. Alternatively, 500 μL of fresh DMEM/F12 was added and they were stored for up to one week at 37°C.

#### Generation of apical-out nasal organoids

Nasal aggregates were generated by first harvesting hNECs expanded in PneumaCult^TM^-Ex Plus medium. Single cells were seeded in 24-well AggreWell^TM^ 400 plates (STEMCELL Technologies, cat # 34411) in various concentrations. Complete PneumaCult^TM^ Apical-Out Airway Organoid medium (AOAOM, STEMCELL Technologies, cat # 100-0620) including heparin (STEMCELL Technologies, cat # 07980) was prepared as per the manufacturer’s instructions. Following seeding of hNECs, the AggreWell^TM^ plate was centrifuged for 3 min at 100 g to sediment and aggregate the cells to the bottom of each microwell. Cultures grown at 37°C were generated with a density of 100 cells per microwell. Cultures that were incubated at 32.5°C had a density of 300 cells per microwell unless indicated otherwise. Similar to the donor optimisation required for apical-out airway organoids, the optimal aggregation time was determined by resuspending aggregates incubated in microwells in 24h intervals. The optimal timepoint was defined as the one where minimal fusion was observed at 24h post suspension. After the aggregates were generated and sufficiently matured, 1 mL fresh medium was added to each well. The aggregates were then resuspended using a P1000 pipette and each well was equally distributed to two wells of a 24-well plate previously treated with Anti-Adherence Rinsing Solution. Resulting aggregates were differentiated in suspension for a total of 15 to 28 days, with a 50% medium change performed every second day. Culture washing was performed by resuspending the culture using a P1000 pipette, allowing approximately one minute for the organoids to sediment to the bottom and subsequently removing 2/3 of the medium together with shed cells. Cultures were imaged using Leica DMi8. To quantify organoid generation efficiency, the total number of organoids generated by a single AggreWell™ well at day 28 was divided by the number of microwells in each well, and this ratio was expressed as a percentage.

#### Generation of nasal ALI cultures and transepithelial electrical resistance (TEER) measurement

Single hNECs were harvested from passage 3 cultures expanded in PneumaCult^TM^-Ex Plus medium and seeded in Costar® 6.5 mm Transwell®, 0.4 μm Pore Polyester Membrane inserts (STEMCELL Technologies, cat # 38024). After submerged culture for 3-5 days in PneumaCult^TM^-Ex Plus to reach confluency, the apical surface was airlifted and the basal medium was changed to complete PneumaCult^TM^-ALI. Media (STEMCELL Technologies, cat # 05001) was changed every other day, and the cultures were assessed as indicated after either 21 or 42 days post airlift. For TEER measurement, media was changed to fresh PneumaCult^TM^-ALI one hour before measuring. TEER was then quantified using EVOM3 (WPI) and following the manufacturer’s instructions.

#### Generation of apical-out airway organoids

Apical-Out Airway Organoids (Ap-O AO) were generated as per the manufacturer’s instructions. Briefly, hBECs were expanded in PneumaCult^TM^-Ex Plus for four passages before harvesting. Single cells were then seeded at a density of 100 cells per microwell into an AggreWell™ 400 plate containing AOAOM. The plates were incubated at either 37°C or 32.5°C at 5% CO_2_, and the cells were allowed to differentiate for 15 days with media changes performed every other day.

#### Immunofluorescence staining

Terminally differentiated organoids were stained and imaged as described previously.^25^ Briefly, apical-out organoids were fixed in Dents fixative (20% Dimethyl sulfoxide (DMSO), 80% methanol) for 3h at room temperature with gentle shaking. Fixed organoids were permeabilised with 1% Triton X-100 (Sigma Aldrich, cat # 10789704001) in Phosphate-buffered saline (PBS), and blocked with 5% Normal Goat serum in PBS supplemented with 0.1% Tween-20 (Sigma Aldrich, cat # P9416) and 0.2% Triton X-100 (PBSTT). Primary antibodies were diluted in PBSTT and incubated with the cells overnight at room temperature in a tube with gentle agitation. Organoids were stained for ZO-1 (Invitrogen, cat #339100) and the epithelial markers acetylated α-TUBULIN (Sigma, cat # T7451), KERATIN 5 (Biolegend, cat # 905501) and MUC5AC (Abcam, cat # ab212636). Goat anti-Mouse IgG2b Cross-Adsorbed Secondary Antibody, Alexa Fluor 568 (Thermo Fisher Scientific, cat # A-21144), Goat anti-Mouse IgG1 Cross-Adsorbed Secondary Antibody, Alexa Fluor 488 (Thermo Fisher Scientific, cat # A-21121) and Donkey anti-Rabbit IgG (H + L), Alexa Fluor 647 (Thermo Fisher Scientific, cat # A-31573) were used respectively as secondary antibodies. Cells were washed with PBSTT and were incubated at room temperature with the respective secondary antibody in a tube with gentle agitation for 1h. Cells were washed again with PBSTT before staining with 4′, 6-diamidino-2-phenylindole (DAPI, Cayman Chemical, cat # 14285) and mounted using SunJin’s RapiClear 1.49 (Scientific Laboratory Supplies, cat # RC149001). ALI cultures were fixed with 100% methanol for 24h at -20°C and then processed similarly to the organoid’s process outlined above. Cultures were imaged using LEICA SP8 or DMi8. To assess the number of goblet cells, individual organoids stained with MUC5AC were imaged and counted manually. Overall, 230 organoids were assessed for Donor 1, 176 for Donor 2 and 218 for Donor 3.

#### Dissociation of apical-out nasal organoids and ALI cultures to perform ciliated cell counts

To dissociate Ap-O NO, cultures were harvested either on day 15 or 28, transferred to a 15 ml tube and centrifuged at 150 g for 5 min. At least 400 organoids were quantified per technical replicate. After an additional spin and removal of the supernatant, they were resuspended in TrypLE Express (Fisher Scientific, cat # 11558856) and incubated for 5-8 min at 37°C, before being dissociated to single cells by pipetting vigorously with a P1000 pipette. For ALI cultures, inserts incubated for either 21 or 42 days were washed with PBS and subsequently submerged in TrypLE Express for 5-8 min at 37°C. Cultures were then triturated with a P1000 pipette to dissociate cells to a single cell suspension. Finally, single-cell suspensions from either method were washed once with DMEM/F12, before diluted 1:1 with Trypan Blue (STEMCELL Technologies, cat # 07050), loaded to a hemocytometer and imaged in Leica DMi8, where the ciliated cells were counted manually.

#### Organoid size, number and cilia presence homogeneity

Fully mature organoids were imaged using a Leica DMi8. Organoid size was measured by imaging mature organoid cultures and using Fiji^49^ to determine the Feret diameter. Organoid number and presence of beating cilia in organoids was manually determined using brightfield microscopy.

#### Quantitative PCR

Total RNA was isolated from Ap-O NO using the Qiagen RNeasy Mini Kit (cat # 74106) following the manufacturer’s instructions. 500 ng of RNA was DNase treated (Invitrogen, cat # AM2222) as per the manufacturer’s protocol and then reverse-transcribed to cDNA using SuperScript III (Invitrogen, cat # 18080-044). TaqMan gene-specific assay primers and probes were obtained from Integrated DNA Technologies (Table 1) and reaction mixture from Applied Biosystems (cat # 4352042). Samples were amplified as follows: denaturation at 95 °C for 3 min followed by 40 cycles at 95 °C for 5 s and 60 °C for 30 s. The mRNA expression levels of cellular genes were normalised with that of *TBP*.

**Table 1:** Accession number of assays used to characterise apical-out nasal organoids

Gene	IDT assay
*KRT5*	Hs.PT.58.14446018
*ITGA6*	Hs.PT.58.453862
*FOXJ1*	Hs.PT.58.40371261
*TUBB4B*	Hs.PT.58.15334509.g
*SCGB1A1*	Hs.PT.58.1190800
*MUC5AC*	Hs.PT.58.24664713.g
*TSLP*	Hs.PT.58.22464960
*FOXI1*	Hs.PT.58.40514934
*TBP*	Hs.PT.39a.22214825(1)

#### RSV infection

Terminally differentiated organoids generated at 32.5°C from hNECs collected from three independent donors were harvested on day 28, washed once with complete PneumaCult^TM^ AOAOM without heparin and seeded at 384-well plates at a density of 100 organoids per cell. Subsequently, organoids were infected with respiratory syncytial virus carrying a mCherry reporter gene (RSV-mCherry^28^) at MOI of 0.001 to 1 in the presence or absence of increasing doses of ribavirin (Merck, cat # R9644). At 24h post-infection, mCherry signal was measured using a BioTek Cytation 5 plate imager (Agilent) and quantified using the GEN5 software. The inhibitory concentrations (IC50) of ribavirin were determined for each donor using GraphPad Prism 10.

### Quantification and statistical analysis

Quantitative data were displayed as means ± s.d. Statistical significances were determined using unpaired t-test, with level of significance set at ∗*p* < 0.05, ∗∗*p* < 0.01, and ∗∗∗*p* < 0.001. Statistical analyses were performed using GraphPad Prism 10.
