# Supplementary material for: Generation of apical-out nasal organoids to facilitate human respiratory syncytial virus infection and drug screening
Source: iScience. 2026 Jul 2;29(7):116638. doi: 10.1016/j.isci.2026.116638 (PMC13355508; doi:10.1016/j.isci.2026.116638)
Supplement: Document S1. Figures S1–S5 and Tables S1–S3 [file mmc1.pdf]

iScience, Volume *29*

Supplemental information

Generation of apical-out nasal organoids to facilitate human respiratory syncytial virus infection and drug screening

Georgios Stroulios, Mathieu Hubert, Wing Chang, Allen Eaves, Sharon Louis, Philipp Kramer, Caroline Tapparel, and Salvatore Simmini

List of Supplementary Information:

Figure S1: Apical-out nasal organoids generated using the conditions optimised for hBECs display suboptimal morphological characteristics. Related to Figure 1.
Figure S2: Modified workflow supports Ap-O NO generation using hNECs from later passages. Related to Figure 2.
Figure S3: Standard culture temperature (37°C) supports more efficient expansion of hNECs compared to 32.5°C. *Related to Figure 2.*
Figure S4: Lower culture temperature impairs differentiation in ALI cultures. Related to Figure 2.
Figure S5: Differentiation at 32.5°C severely impacts apical-out airway organoid morphology Related to Figure 2.

Table S1: Ciliated cell counts relating to Figure 1F

Table S2: Ciliated cell counts relating to Figure 2B

Table S3: Ciliated cell counts relating to Supplementary Figure 2B

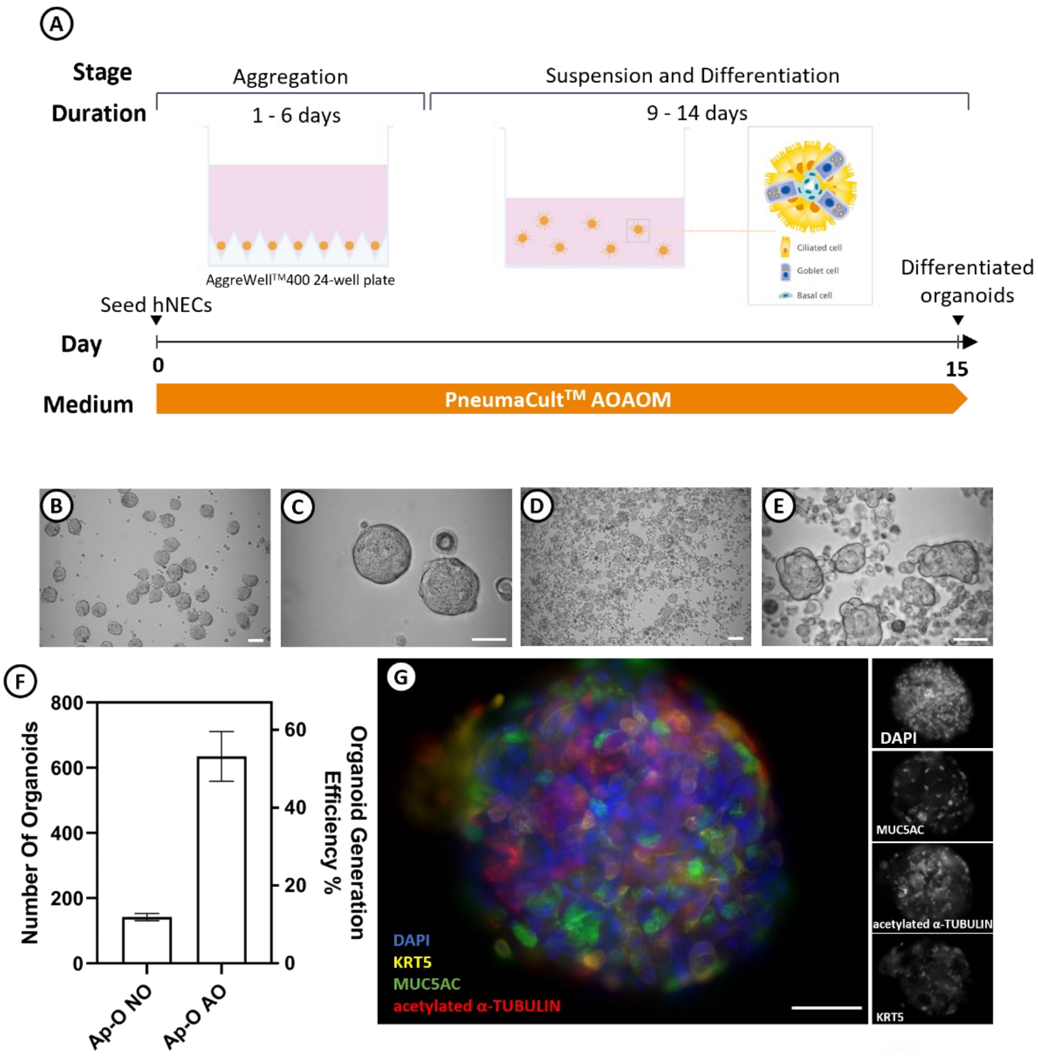

Figure S1: Apical-out nasal organoids generated using the conditions optimised for hBECs display suboptimal morphological characteristics. Related to Figure 1.
A. Schematic outline of the protocol used to generate apical-out nasal organoids.
B-C. Representative brightfield image of cultures at day 1 at low (B, scale bar = 100 µm) and high (C) magnifications. (Scale bars = 50 µm, n = 1 donor)
D-E. Representative brightfield image of cultures at day 15 at high (C, scale bar = 100 µm) and low (D, scale bar = 50 µm) magnifications.
F. Graph illustrating the average number (± standard deviation) of Ap-O NO and Ap-O AO obtained from a well of an AggreWell™ 400 24-well plate at day 15 using p3 hNECs and hBECs respectively. (n=1 donor for Ap-O NO and n=3 donors for Ap-O AO).
G. Whole mount ICC stain of organoids at day 15 imaged with a fluorescent microscope. Markers of basal (KRT5, yellow), ciliated (acetylated α-TUBULIN, red) and goblet (MUC5AC, green) cells can be identified. On the right, each channel is presented separately in grey channel. (Scale bar = 50 µm, n = 1 hNEC donor).

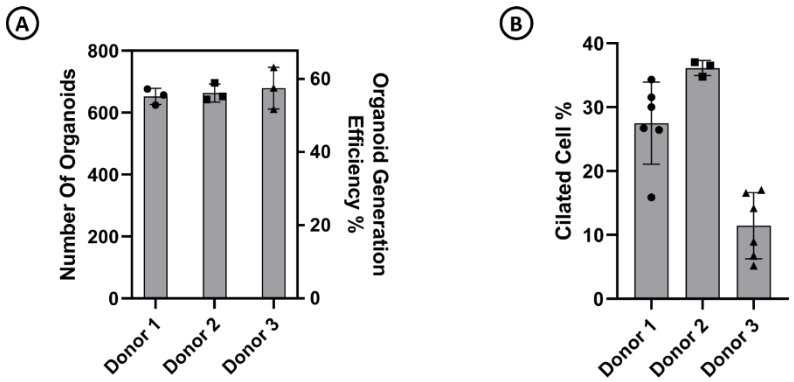

Figure S2: Modified workflow supports Ap-O NO generation using hNECs from later passages. Related to Figure 2.

A. Number of generated organoids per well of a 24-well tissue culture plate across three different donors generated from p4 hNECS. Data are presented as mean ± SD, and points represent independent wells of a 24-well plate. (n = 3 donors)
B. Ciliated cell percentage of generated apical out organoids at day 28 generated from p4 hNECs. Data are presented as mean ± SD, and points represent measurements taken from independent wells of a 24-well plate. (n = 3 hNEC donors)

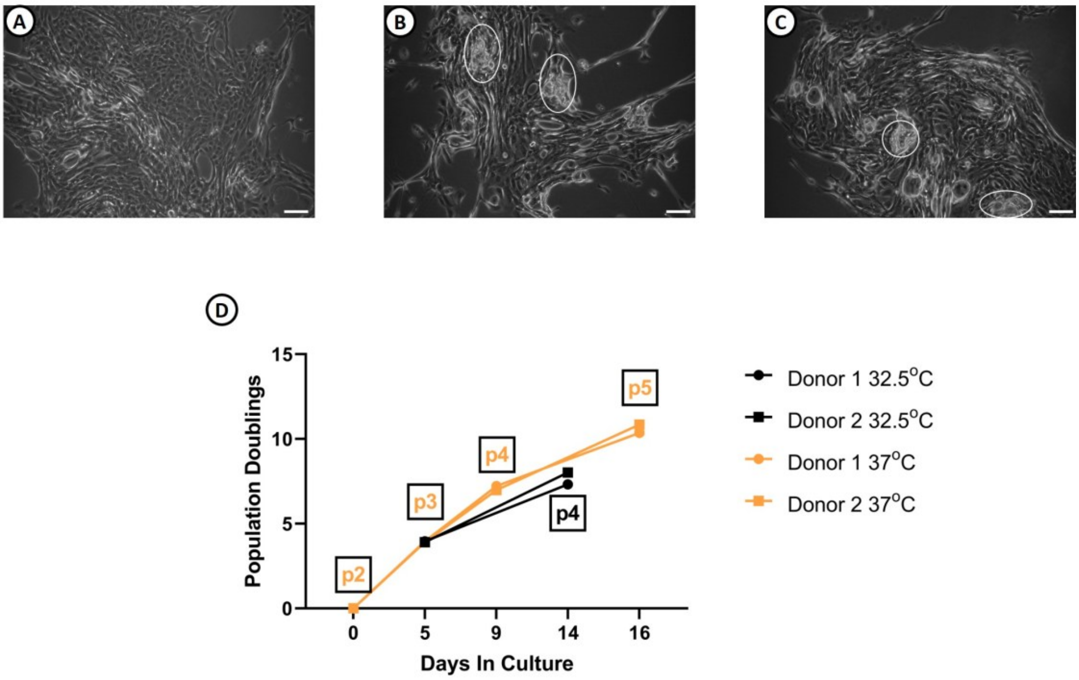

Figure S3: Standard culture temperature (37°C) supports more efficient expansion of hNECs compared to 32.5°C. Related to Figure 2.

A-C. Representative images and growth data for hNEC cultures from 2 donors. A confluent p3 culture grown at 37°C, imaged on day 4 immediately before passaging (A). A p4 culture grown at 37°C, imaged on day 7 (B). A p3 culture grown at 32.5°C, imaged on day 9 (C). White circles in (B) and (C) indicate cells with a "bubbly" morphology. (Scale bars = 100 μm)
D. Cumulative population doublings for cultures maintained at 37°C (black points) and 32.5°C (orange points). Individual points mark passages and the adjacent square indicates the passage number. Points represent average ± standard deviation of 2 technical replicates per condition.

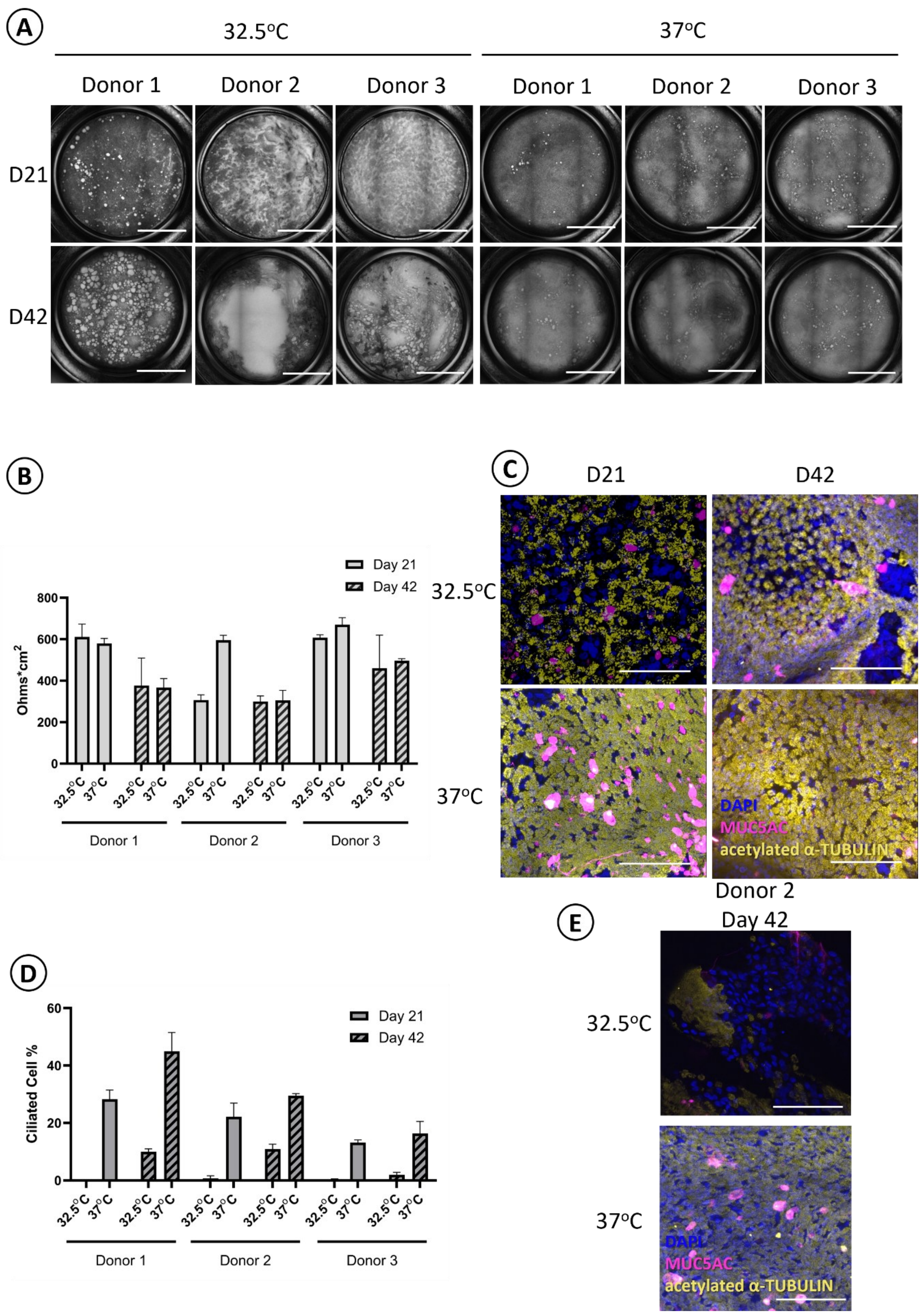

Figure S4: Lower culture temperature impairs differentiation in ALI cultures. Related to Figure 2.

A. Representative brightfield images of 3 hNEC donors cultured at 32.5°C or 37°C for 21 and 42 days. (Scale bars = 2.5 mm)

B. TEER measurements of ALI cultures generated at 32.5°C or 37°C and assessed on day 21 or 42. Points represent average ± standard deviation of 3 technical replicates per condition.
C. Immunofluorescence staining of ALI cultures from Donor 1 for the ciliated cell marker acetylated a-TUBULIN (yellow) and the goblet cell marker MUC5AC (magenta) at days 21 and 42. Nuclei are counterstained with DAPI (blue). (Scale bars = 100 µm)
D. Ciliated cell percentage of ALI cultures generated at 32.5°C or 37°C and assessed on day 21 or 42. Points represent average ± standard deviation of 3 technical replicates per condition.
E. Immunofluorescence staining of ALI cultures from Donor 2 for the ciliated cell marker acetylated a-TUBULIN (yellow) and the goblet cell marker MUC5AC (magenta) at day 42, at 32.5°C or 37°C. Nuclei are counterstained with DAPI (blue). (Scale bars = 100 µm)

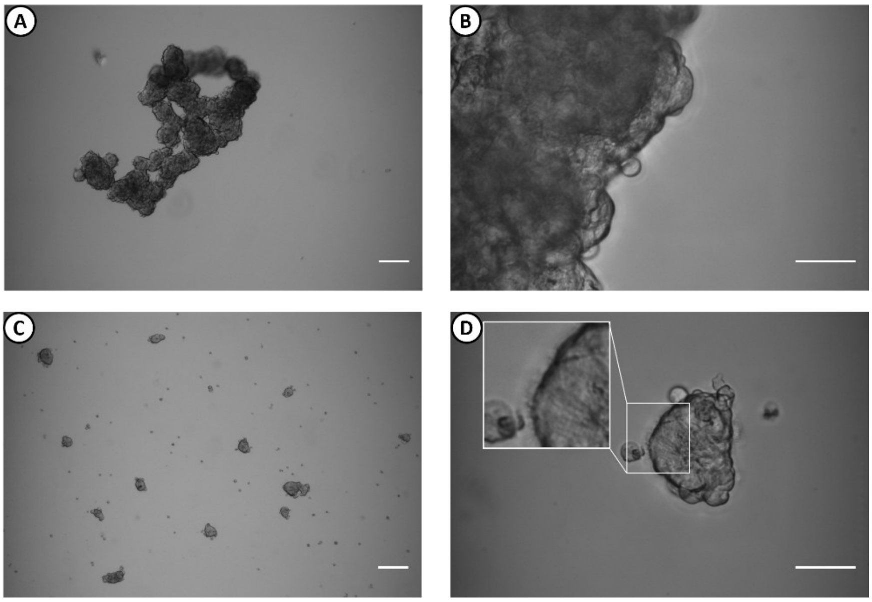

Figure S5: Differentiation at 32.5°C severely impacts apical-out airway organoid morphology. Related to Figure 2.

A-D. Representative brightfield images of cultures from 2 hBEC donors on day 15. At 32.5°C, cultures display extensive fusion into a large aggregate (A). A higher-magnification view of the aggregate edge from panel A (B). In contrast, cultures maintained at 37°C show minimal fusion (C). A magnified view of an organoid grown at 37°C reveals beating cilia on its outer surface (D). Scale bars = 200 µm (A, C) and 50 µm (B, D).

Day 15		
	Ciliated Cells	Total Cells
Organoids at 32.5°C	6	337
	4	371
Organoids at 37.5°C	31	72
	82	144
Day 28		
	Ciliated Cells	Total Cells
Organoids at 32.5°C	119	342
	134	367
Organoids at 37.5°C	n/a	n/a
	n/a	n/a

Table S1: Ciliated cell counts relating to Figure 1F

	32.5°C		32.5°C	
	Ciliated Cells	Total Cells	Ciliated Cells	Total Cells
Donor 1	121	234	145	460
	136	315	150	378
	169	325	211	522
	172	324		
Donor 2	62	174	123	503
	133	322	200	731
	114	256	261	843
	97	262		
Donor 3	21	220	5	313
	11	141	4	269
	32	306	7	257
	24	184		

Table S2: Ciliated cell counts relating to Figure 2B

	Ciliated Cells	Total Cells
Donor 1	93	348
	96	363
	140	408
	139	463
	47	296
	117	371
Donor 2	225	645
	134	367
	187	505
Donor 3	29	170
	29	175
	45	317
	25	279
	11	163
	9	174

Table S3: Ciliated cell counts relating to Supplementary Figure 2B
